# Mild temperatures differentiate while extreme temperatures unify gene expression profiles among populations of *Dicosmoecus gilvipes* in California

**DOI:** 10.3389/fphys.2022.990390

**Published:** 2022-10-05

**Authors:** Emily E. King, Jonathon H. Stillman

**Affiliations:** ^1^ Department of Integrative Biology, University of California, Berkeley, Berkeley, CA, United States; ^2^ Department of Biology, San Francisco State University, San Francisco, CA, United States

**Keywords:** gene expression, caddisfly, thermal, ectotherm, warming, local adaptation, acclimation, acclimatization

## Abstract

Accurately predicting the effects of future warming on aquatic ectotherms requires an understanding how thermal history, including average temperature and variation, affects populations of the same species. However, many laboratory studies simplify the thermal environment to focus on specific organismal responses and sacrifice environmental realism. Here, we paired laboratory-based transcriptomic RNA-seq analysis to identify thermally responsive genes with NanoString analysis of a subset of those genes to characterize natural field-based variation in thermal physiology among populations. We tested gene expression responses of three populations of field-acclimatized larval caddisflies (*Dicosmoecus gilvipes*) from streams in different eco-regions (mountain, valley, and coast) following exposure to current and future summertime temperatures. We hypothesized that distinct thermal histories across eco-regions could differentiate populations at baseline “control” levels of gene expression, as well as gene expression changes in response to daily warming and heat shock. Population-specific patterns of gene expression were apparent under the control and daily warming conditions suggesting that local acclimatization or local adaptation may differentiate populations, while responses to extreme temperatures were similar across populations, indicating that response to thermal stress is canalized. Underlying gene co-expression patterns in the daily warming and heat shock treatments were different, demonstrating the distinct physiological mechanisms involved with thermal acclimatization and response to thermal stress. These results highlight the importance and limitations of studies of the thermal biology of wild-caught organisms in their natural environment, and provide an important resource for researchers of caddisflies and aquatic insects in general.

## Introduction

Predicting the responses of aquatic ectothermic animals to changing temperature regimes is critical for managing biodiversity in the future. Temperature influences many vital aspects of physiology, development, and life history, and ultimately shapes biogeography (Sweeny et al., 1992; Gillooly et al., 2001; Seebacher et al., 2015). Animals can deal with changes in temperature at different time and biological scales. At the organismal level, animals can acclimate or adjust their physiology after long-term exposure to a thermal stimulus (days to weeks) (Bowler, 2005; Angilletta, 2009; Shah et al., 2017). Organisms from more variable environments are expected to have a greater capacity for acclimation than organisms in stable environments (Shah et al., 2017). Warm acclimated individuals are expected to have higher thermal tolerances than cold-acclimated individuals. In freshwater organisms specifically, this results in decreased thermal sensitivity of organismal traits to further warming for already warm acclimated populations (Seebacher et al., 2015). At the biochemical level, organisms can change the regulation of gene expression in response to thermal exposures. Specifically, expression of heat shock proteins and other molecular chaperones, macromolecules that quickly protect damaged or denaturing proteins, is a reliable indicator of rapid temperature changes (over hours) (Feder and Hoffman, 1999; Dahlhoff, 2004; Somero, 2005; Somero, 2010).

Thermal history is made up of both average temperatures and thermal variation and can lead to sustained differences in physiological responses between environments or populations. Protein expression of aquatic stonefly species in Japan differs along stream temperature gradients that covary with latitude and elevation. Protein expression was more similar between species with shared thermal history than within a species across regions (Gamboa et al., 2017). Acclimation to different thermal histories can also cause warm-acclimated populations to induce heat shock protein expression at higher temperatures than cold-acclimated populations (Bowler, 2005). What may appear as a muted response to a temperature change in one population may indicate a warmer thermal history. Thermal history plays a critical role in our ability to forecast future responses to temperature change in wild populations.

Physiologists often acclimate animals to constant temperatures before an experiment and use “average” conditions as the experimental treatments (e.g., average summer temperature vs. average winter temperature) (Morash et al., 2018). In these highly controlled lab studies, we can focus on responses to a particular stimulus without certain complexity (e.g., individual variation, temperature variation), but this simplicity comes at the cost of biological realism. Stable laboratory conditions mask thermal history and rarely elicit responses similar to those measured under fluctuating conditions (Denny, 2017; Morash et al., 2018; Marshall et al., 2021). One solution is to study animals in their natural, variable environments to understand how thermal history affects the response of different populations. To make the best conclusions about future changes in biogeography and population dynamics under future warming conditions, we need to make population-level inferences under realistic conditions (Pörtner and Knust, 2007; Dong et al., 2015; Morash et al., 2018).

Caddisfly larvae are an integral element of California river ecosystems. *Dicosmoecus gilvipes* occur in various eco-regions of California, including mountain, valley, and coastal populations experiencing different thermal means and variabilities. Mountain streams freeze over then warm dramatically through the summer, while coastal streams stay within a much warmer, narrower temperature range. Future warming is likely to raise the maximum water temperatures in all eco-regions (Null et al., 2013). Temperature has clear effects on the physiology and life history of *D. gilvipes*. Larvae develop faster in stream regions with a higher number of degree days until they disappear in the most downstream regions (Hannaford, 1998; Resh et al., 2011). There is low gene flow between populations due to a short-lived adult stage (∼2 weeks) and short-range mating cues (Resh and Wood, 1985; Peterson et al., 2017), thus separating populations genetically and by thermal environment. Therefore, *D. gilvipes* is a good model for understanding how temperature warming will impact wild, free-living aquatic animals with different thermal histories.

This study tests the hypotheses that wild populations of larval *D. gilvipes* will differ in baseline gene expression and responses to current and future warming scenarios due to differences in thermal history. Specifically, we expect that 1) populations from warm locations will have less thermally sensitive gene expression responses, 2) that the more extreme warming scenario will generate more extreme transcriptional responses, 3) that long and short summer acclimatization will generate different transcriptional responses, and that 4) gene co-expression will be similar between warming scenarios. To test these hypotheses, we assayed the expression of thermally sensitive mRNA transcripts in three populations of field acclimated *D. gilvipes* from three eco-regions at two dates through the summer.

## Materials and methods

### Animal collection and thermal exposure

**FIGURE 1:**
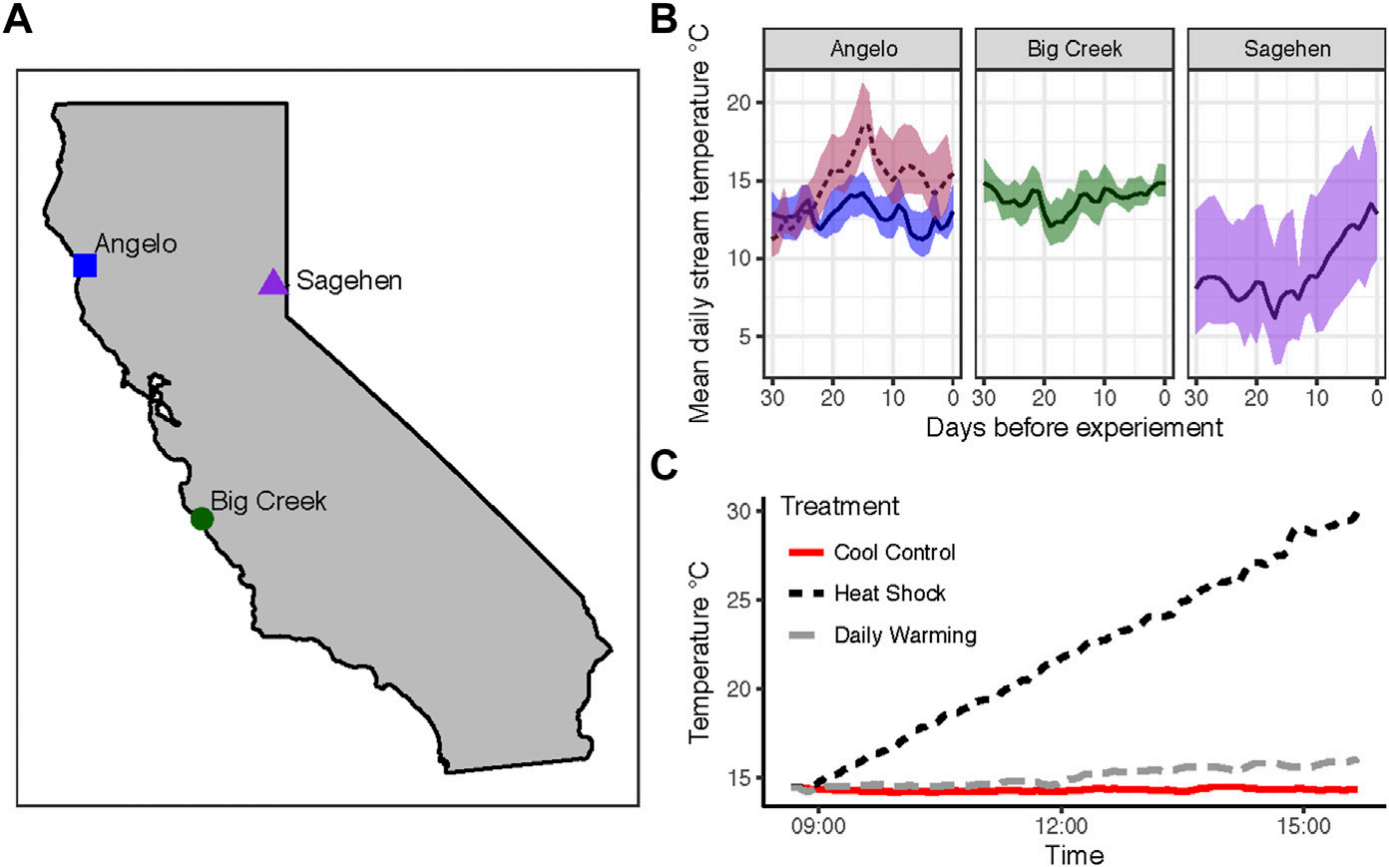
Thermal histories and experimental exposures of larval *Dicosmoecus gilvipes* caddisflies **(A)** Three California populations used in this study and **(B)** stream temperatures at each of these sites in the 30 days before the experiment. The data represent the following dates from each site: Angelo (4/29/13–5/29/13 and 5/24/13–6/22/13) in blue and pink, respectively, Big Creek (5/10/13-6/9/13) in green, and Sagehen (5/12/13–6/11/13) in violet. The solid line represents the mean daily stream temperature. The shaded area around the line indicates the daily temperature range **(C)** Example of experimental temperature treatments from Big Creek (6/9/13). Colors and line types represent the warming treatment.

We collected larval *Dicosmoecus gilvipes* (Hagen, 1875) from three distinct populations at stream sites within the University of California Natural Reserve System. Larvae in the fifth (terminal) instar stage were collected by hand in the morning on the day of each experiment from Angelo Coast Range Reserve (39.7186°, −123.6528°), Sagehen Creek Reserve (39.4333°, −120.2407°), and Landels-Hill Big Creek Reserve (36.071298°, −121.599153°) (Figure 1A). Larval stage was determined by case-building materials (Resh et al., 2011; Holomuzki et al., 2013). Thermal stress experiments were performed streamside May-June of 2013 (Table 2). The experiment was run twice at the Angelo site (i.e., “Angelo early,” “Angelo late”) just under 1 month apart to measure the effects of seasonal acclimatization on the same population. Stream temperatures for the 30 days preceding each experiment were obtained from stream sensor data collected by other researchers (Table 1; Figure 1B). These sites represent three temperature regions in California: mountain (Sagehen, elevation 1972m), valley (Angelo), and coastal (Big Creek).

**TABLE 1:** Stream temperatures in the field for 30 days before collection (°C).

Site	Avg. Daily Temp	Avg. Daily Max	Avg. Daily Min	Avg. Daily range	Absolute Max	Absolute Min	Source	Date range
Angelo	12.8	14.0	11.7	2.4	15.6	10.1	Berkeley Sensor Database^a^	4/29–5/29/2013
Big Creek	13.8	15.0	12.8	2.2	16.4	10.9	NOAA Fisheries, Santa Cruz^b^	5/10-6/9/2013
Sagehen	9.19	13.9	6.1	7.9	18.5	3.2	USGS NWIC^c^	5/12–6/11/2013
Angelo	14.9	17.0	13.6	3.4	21.2	10.1	Berkeley Sensor Database^a^	5/24–6/22/2013

a http://sensor.berkeley.edu/cgi/sensor_query2?view=check_password&Access=4&username=Guest+User&MC_Name=Angelo+Reserve&VariableCode=Water+Temp+C&ONEVAR=1.

b Personal communication with Dave Rundio.

c https://waterdata.usgs.gov/ca/nwis/uv/?site_no=10343500&PARAmeter_cd=00065,00060.

Three to four individual caddisflies were held in continuously aerated 1 L glass jars inside one of three insulated water coolers to maintain specific temperatures (Table 2). We collected a small number of individuals for each treatment due to the availability of appropriate larval stage on the experimental dates. Cooler temperatures were manipulated to represent three temperature treatments: a cool control, gradual warming mimicking the daily increase in stream temperature, or a heat shock to 30°C. We will refer to the treatments as “cool control,” “daily warming,” and “heat shock.” The cool control treatment was held at the temperature of the stream at the time the caddisflies were collected in the morning (Table 2; Figure 1C) for the duration of the experiment, approximately 9 h. The daily warming treatment matched the warming stream through the day by measuring the temperature of the stream every 2 minutes with an Omega HH603A handheld thermometer. The temperature of the treatment water was manipulated manually by adding hot water or bags of ice into the outer chamber of the cooler to achieve a temperature matching the stream. Each site had a slightly different natural warming profile, but endpoint temperatures were between 15° and 17°C (Table 2). The heat shock treatment water was warmed from the control temperature to 30°C at a rate of approximately 3 C/h. The maximum temperature, 30°C, reflects the annual maximum temperature observed at the Angelo Coast Reserve plus a 4°C warming based on end-century climate change predictions (Hannaford, 1998; IPCC, 2019). After a 1-h exposure to the maximum temperature of the respective treatment, individuals were removed from their case, blotted dry, flash-frozen in liquid nitrogen, and stored at −80°C prior to RNA extraction. There were no mortalities in any treatment at any site.

**TABLE 2:** Experimental temperatures treatments (°C) and number of individuals analyzed in parentheses.

Site	Experiment date	Control	Daily warming	Heat shock
Angelo early	29 May 2013	12°(3)	12°–15°(3)	12°–30°(4)
Sagehen	9 June 2013	13°(4)	13°–17°(4)	13°–30°(3)
Big Creek	11 June 2013	14°(4)	14°–16°(4)	14°–30°(3)
Angelo late	22 June 2013	14°(4)	14°–17°(4)	14°–30°(2)

### Selection of biomarkers

Target genes were chosen for NanoString analysis from among the most differentially expressed genes in a laboratory temperature exposure RNA-Seq experiment (Supplementary Material; Supplementary Figures S1, S2; Supplementary Table S1) ([*D. gilvipes* transcriptomic response] Stillman, 2022). Reference candidate genes were selected based on the lowest coefficient of variation in FPKM (fragments per kilobase of exon per million fragments mapped) values from the same RNA-Seq experiment with a range of expression levels and reasonable biological function (i.e., transcription apparatus, cytoskeleton). Thirty-one NanoString targets were selected, representing both those that increased and decreased with warming, to be normalized to the three reference genes described above (Supplementary Table S2).

### RNA preparation

Head and thorax tissues were homogenized with stainless steel ball bearings (3 mm, McMaster Carr) in Tri Reagent (Molecular Research Center, United States) using a TissueLyser II (Qiagen). RNA was isolated according to the manufacturer’s recommended protocol, using bromochloropropane (BCP, Molecular Research Center, United States) for phase separation and isopropanol for RNA precipitation (Chomczyński and Sacchi, 1987). RNA quality and quantity were measured with a Bioanalyzer (Agilent). Only samples with little to no degradation and adequate concentration were used in downstream steps.

DNA-free RNA was prepared by mixing 5 µg of RNA, 5 µl of 10x reaction buffer (Thermo Scientific, 100 mM Tris-HCl, 25 mM MgCl_2_, 1 mM CaCl_2_) and 5 U of DNase I enzyme (Thermo Scientific). RNase-free water was added to a final volume of 55 µl. The DNase reaction proceeded at 37°C for 30 min and was stopped by adding 5 µl of 50 mM EDTA with heating at 65°C for 10 min. The DNA-free RNA was stored at −80°C.

### NanoString expression and data quality control

Gene expression was measured using the nCounter System (NanoString Technologies) (Geiss et al., 2008). Some of the RNA extracted from each caddisfly sample was diluted with RNase-free water to a concentration of 20 ng/μl in a final volume of 20 µl. Samples were then sent to NanoString Technologies in Seattle, WA, USA for processing and gene expression quantification. This method allows for many genes to be analyzed at once without cDNA reverse transcription or amplification to reduce bias in counts of rare or abundant transcripts. The resulting expression data were background corrected by subtracting the mean plus two standard deviations of the negative controls. Transcripts with post-background correction expression values in the negative range were excluded. Expression levels of the remaining transcripts were normalized to the geometric mean of the three reference genes for that individual and log-transformed.

### Data analysis

All analysis was completed in the R Computing Environment (R Core Team 2018). Principal components analysis using the “pcaMethods” package in R (v1.72.0, Stacklies et al., 2007) was used to identify potential population-level differences in the entire suite of genes. The data were mean-centered and scaled using the Pareto scaling method prior to running the principal components analysis. The Pareto method scales the data with the square root of the standard deviation (Eriksson et al., 1999). We tested for statistical differences in PC scores between groups using a MANOVA with the Wilks *λ* test statistic. One analysis compared the first sampling date at all sites and the second analysis compared the two sampling dates from Angelo (early and late). The model structures were: PC1+PC2∼Population*Treatment and PC1+PC2∼Date*Treatment. We ran univariate ANOVAs on the significant components of the MANOVA to do Tukey’s multiple pairwise comparisons (Supplementary Table S2). We excluded one individual (from Sagehen control) from the PCA analysis because inspection of residuals suggested that it was a multivariate outlier and thus violated the assumptions of MANOVA (Supplementary Figures S3, S4). The overall conclusions did not change when this outlier was excluded, and normality of residuals was improved (Supplementary Figure S4).

To focus on the responses to the two warming scenarios, we analyzed the change in expression between the cool control and each of the warming treatments. For each transcript, the average expression level for that population under control conditions was subtracted from each individuals’ warming response to give Δ expression, separately for daily warming and heat shock. The resulting positive values indicate increased expression under a warming treatment, while negative values indicate decreased expression relative to the control. This procedure was performed for the daily warming treatment and the heat shock treatment. Comparisons between sites within one warming treatment and between warming treatments were made using Kruskal-Wallis tests and Nemenyi post-hoc tests to handle non-parametric datasets.

Heatmaps were created with “heatmap3” (v1.1.9, Zhao et al., 2021). Dendrograms clustered rows of genes by similarity of Δ expression from the treatment control. The colors of the heat map cells represent the magnitude and direction of the change in expression, scaled and centered by row.

Gene co-expression matrices were created for each warming treatment using “corrplot” (v0.84, Wei and Simko 2016). Matrix data were Pearson’s correlations of gene expression in each treatment and were ordered by gene function. The threshold for a significant correlation was *α* = 0.05. Populations were combined to provide a sufficient sample size for correlation analysis. Clusters of important genes were identified by visual inspection.

## Results

### Thermal history

In the month preceding the first round of sampling, the stream temperature was very similar at Angelo (valley) and Big Creek (coastal) (Table 1; Figure 1B). Stream temperature at Sagehen, a montane site, is characterized by much lower minimum temperatures and a much larger daily temperature range. At Sagehen, there was also a sustained warming trend in the 10 days before the experiment (Figure 1B). A few days before the experiment, maximum daily water temperatures exceeded the temperature of the daily warming treatment for that population (Tables 1, 2). The mean temperature preceding the Angelo “late” sampling was 2°C warmer with a wider range and higher maximum temperature relative to the Angelo “early” sampling. Fifteen days before the Angelo “late” sampling time, maximum temperatures were higher than any daily warming treatment and were only exceeded by the heat shock treatments (Table 1).

### Gene expression differentiates populations and treatments

**FIGURE 2:**
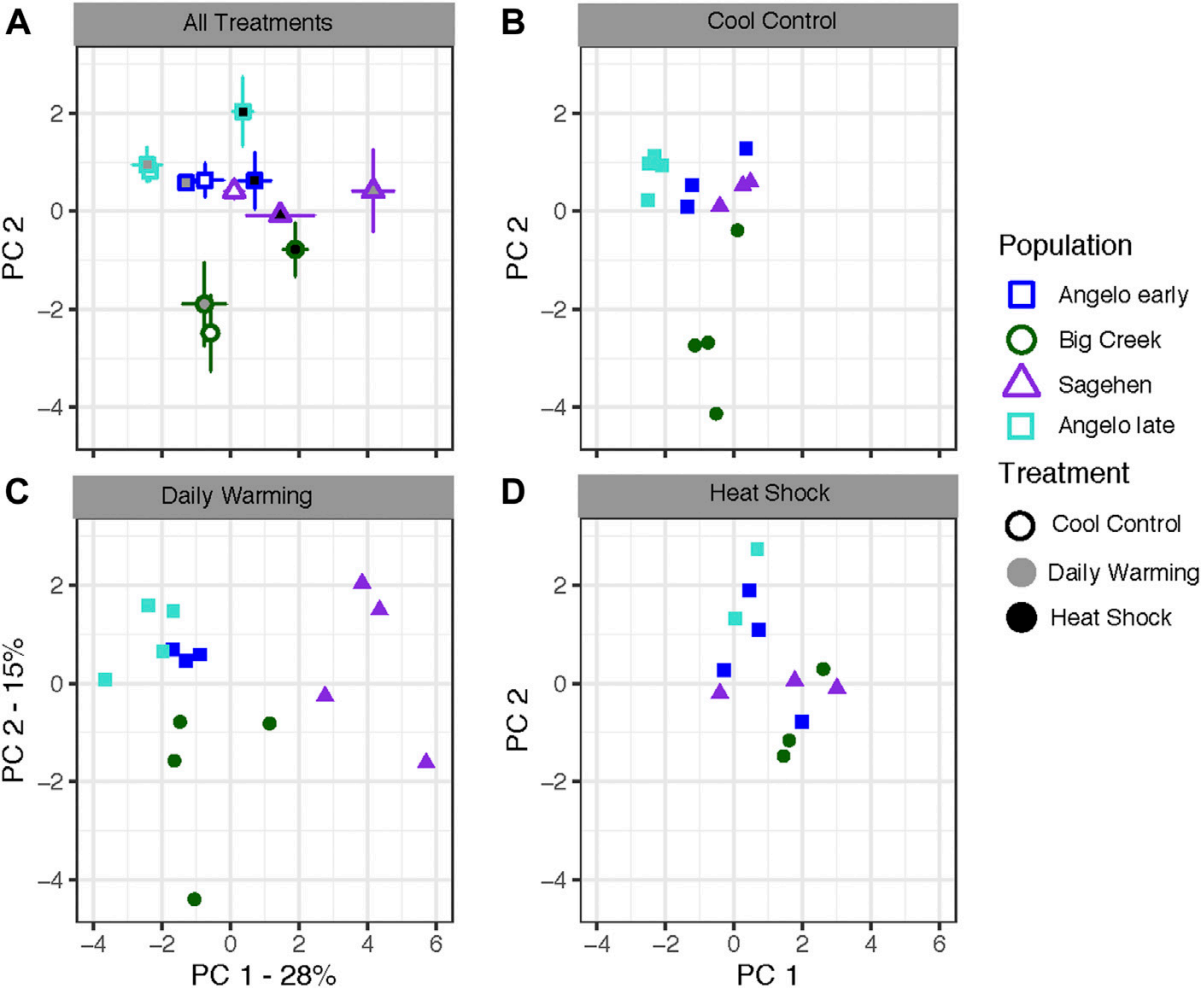
Gene expression analysis of *Dicosmoecus gilvipes* caddisflies exposed to three different temperature treatments. PC 1 represents 28% of the variation, while PC 2 represents 15% of the variation. **(A)** Mean PC scores for all population and treatment combinations. Points represent means with standard error bars along both axes. Point outlines represent population of origin, fill color represents the treatment **(B–D)** Individuals plotted by their scores on PC1 and PC2 in the **(B)** cool control treatment, **(C)** daily stream warming treatment, and **(D)** heat shock treatment. This analysis removed one outlier point from Sagehen control.

Population, treatment, and their interaction had significant effects on PCs 1 and 2 for the first sampling time from all populations (Figure 2A; Tables 3A,B). PC1 represented 28% of the variation in expression, while PC 2 represented 15% of the variation. Differences along PC1 were significantly positively correlated with 4 of 5 heat shock proteins in our study (Supplementary Figure S5). PC1 was negatively correlated with *apoptosis inhibitor* and *circadian clock protein*. PC 2 was positively correlated with a suite of genes containing metabolic genes and molecular transporters, among genes of other functions. Broadly, all treatments in the same populations grouped together (Figure 2A). Angelo typically had low PC1 scores and high PC2 scores. Big Creek had significantly lower PC2 scores than the other populations (Supplementary Table S3B). Sagehen had medium to high PC1 scores significantly different from the other populations (Supplementary Table S3A). When the treatments are compared separately, they group by population in the control and daily warming treatments, but not in the heat shock treatment (Figures 2B–D).

**TABLE 3A:** Effect of population and date on principal component scores of temperature-sensitive transcripts in *Dicosmoecus gilvipes.* Results of MANOVA tests to determine the effects of population on the early experiments.

	df	Wilks *λ*	F	Num df	Den df	*p*
Population	2	0.18238	14.0869	4	42	**2.27e-07**
Treatment	2	0.57752	3.3168	4	42	**0.019**
Population*Treatment	4	0.33791	3.7815	8	42	**0.002**
Residuals	22					

Significant results are bolded.

**TABLE 3B:** Effect of population and date on principal component scores of temperature-sensitive transcripts in *Dicosmoecus gilvipes.* Results of MANOVA tests to determine the the effect of the additional warming between the two Angelo dates.

	df	Wilks *λ*	F	Num df	Den df	*p*
Date	1	0.34354	12.4207	2	13	**0.001**
Treatment	2	0.28410	5.6949	4	26	**0.002**
Date*Treatment	2	0.77925	0.8634	4	26	0.499
Residuals	14					

Significant results are bolded.

We compared the early and late sampling times from Angelo to understand if gene expression responses to warming change with seasonal acclimatization during summer warming. The two sampling dates and the treatments significantly differed along PC1, but there was no differentiation on PC2 (date: *p* < 0.001, treatment: *p* < 0.01) (Table 3B). The Angelo “late” samples generally had lower scores on PC1 compared to the Angelo “early” samples. The daily warming treatment did not differ from the control treatment, but all other treatments were distinct from each other (Supplementary Table S3).

### Response to warming is site and treatment specific

**FIGURE 3:**
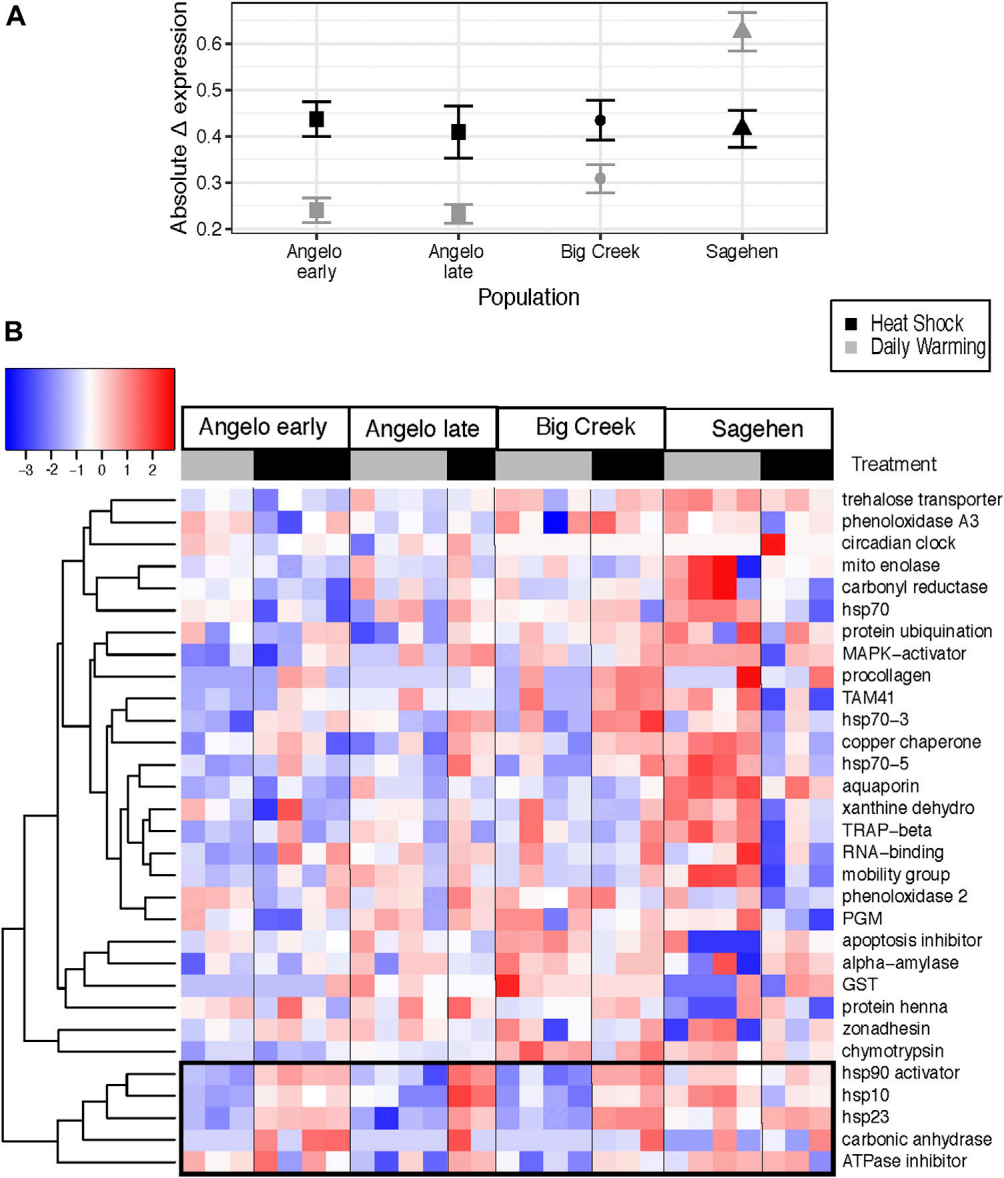
Change in expression between cool control and warming treatments for *Dicosmoecus gilvipes* from three California streams **(A)** Mean ± standard error of the absolute change in expression (Δ expression) from the control at each site and treatment. Gray points represent the daily stream warming treatment, and black points represent the heat shock treatment. **(B)** Heatmap of difference in gene transcript abundance relative to the cool control treatment. Genes are arranged in rows and grouped by similarity of induction value (dendrogram). Each column is an individual caddisfly, labeled by its warming treatment. Black and grey bars correspond to the daily warming treatment and heat shock, respectively. The colors of the heat map cells represent the magnitude and direction of the change in expression, scaled and centered by row. The black box highlights a cluster of genes with similar expression patterns (See Figure 4).

The magnitude of changes in gene expression relative to the control treatment was larger in the heat shock treatment than the daily warming treatment (Figure 3A) (Kruskal-Wallis test, χ^2^ = 6.3835, *p* < 0.05). At Sagehen, Δ expression in the daily warming treatment is more than two times higher than at Angelo (Kruskal-Wallis test, *p* < 0.05, Table 4A). Δ expression in the heat shock treatment did not differ across populations (Kruskal-Wallis test, *p* > 0.05).

**TABLE 4A:** Effect of population and treatment on gene expression responses to warming results of Kruskal-Wallis test to determine population differences for absolute Δ expression of genes in the daily warming treatment and the Nemenyi post-hoc test.

Kruskal-Wallis test			
	χ^2^ = 60.317	df: 3	**p < 0.001**
Post-hoc comparisons using Nemenyi test			
	Angelo early	Angelo late	Big Creek
Angelo late	0.9988	—	—
Big Creek	0.0727	**0.0330**	—
Sagehen	**3.8e-07**	**4.0e-10**	**0.0064**

Significant results are bolded.

**FIGURE 4:**
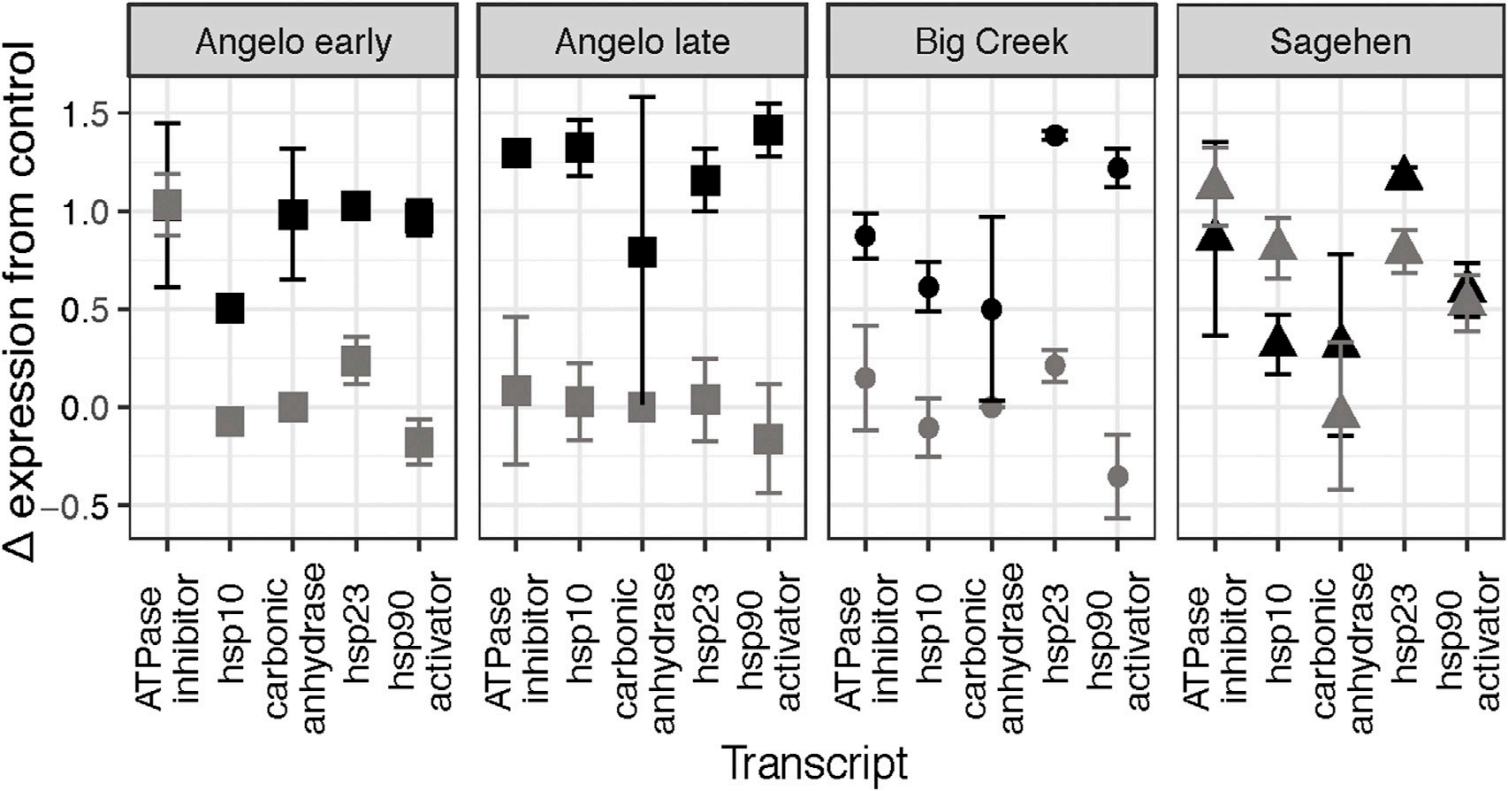
Change in expression relative to the control treatment for the five genes clustered in the box in Figure 3B for each site. The daily warming treatment is represented in gray, and the heat shock treatment is represented in black.

Five genes were clustered together with the most similar Δ expression profiles (Figure 3B). Three genes in that cluster are molecular chaperones (hsp10, hsp23, hsp90 activator), and the others are related to metabolism (ATPase inhibitor, carbonic anhydrase). There are significant differences in Δ expression with the main effects of population, treatment, and their interaction (MANOVA, Table 4B). Within that cluster, gene expression was generally up-regulated to a greater degree in response to heat shock than daily warming at Angelo and Big Creek, with little or no up-regulation of gene expression in response to daily warming (Figure 4). In contrast, Sagehen individuals up-regulated expression of these genes to similar levels in both warming treatments (Figure 4).

**TABLE 4B:** Effect of population and treatment on gene expression responses to warming results of MANOVA to determine the effects of population and treatment on the expression of genes grouped together in Figures 3, 4.

	df	Wilks *λ*	F	Num df	Den df	*p*
Population	3	0.15806	2.6503	15	41.81	**0.007**
Treatment	1	0.09988	27.0362	5	15	**<0.001**
Population*Treatment	3	0.15948	2.6327	15	41.81	**0.007**
Residuals	19					

Significant results are bolded.

### Gene co-expression differs between warming treatments

**FIGURE 5:**
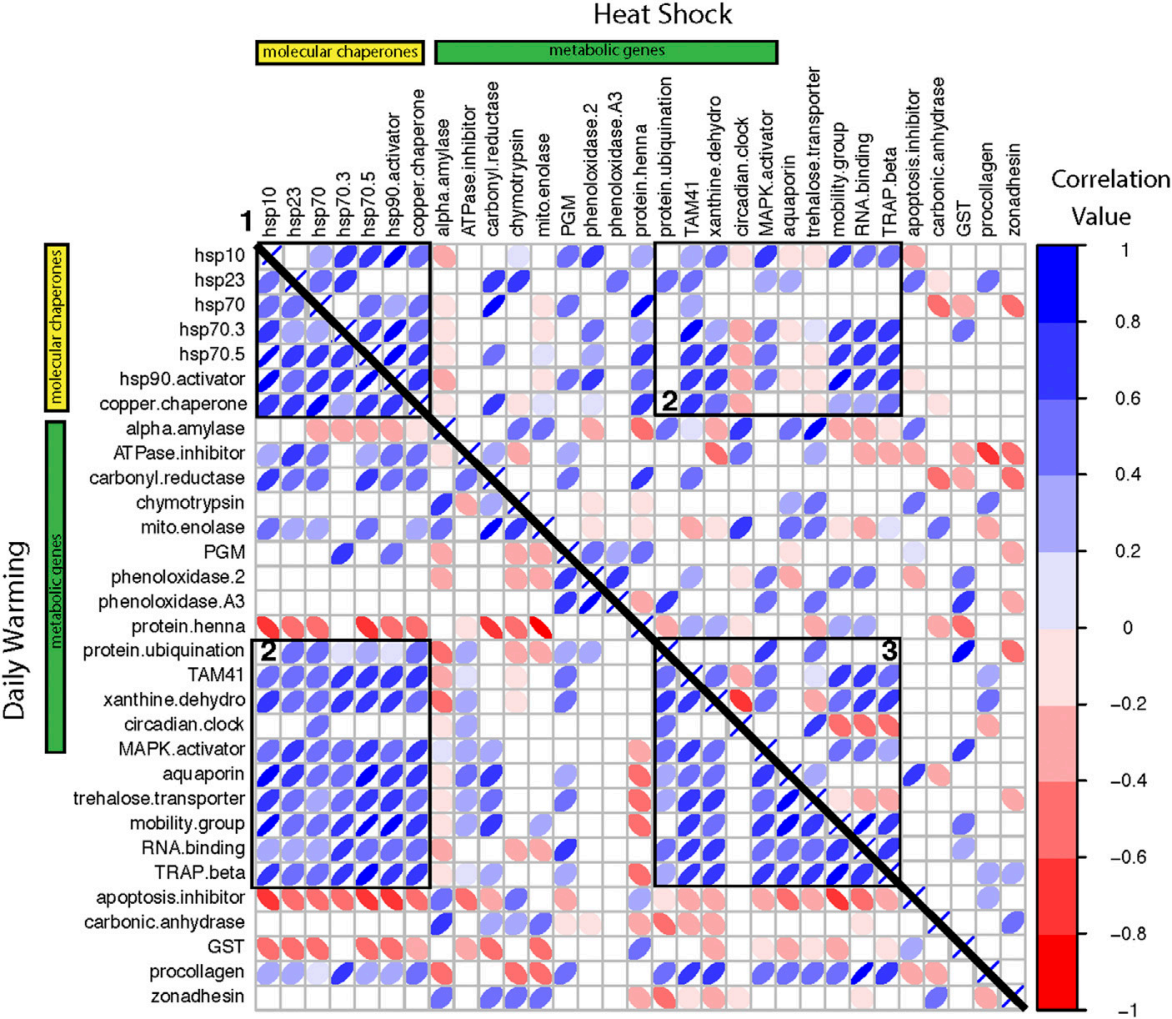
Correlation matrices of gene expression change ordered by functional category for the daily warming treatment on the lower triangle and the heat shock treatment on the upper triangle. The yellow bar indicates molecular chaperones. The green bar indicates metabolic genes. The remaining genes have various functions (see Supplementary Table S2). Each row/column is one gene-by-gene comparison. The color and width of the ellipse indicate the correlation strength and direction. Blank cells represent non-significant correlations. Numbered boxes denote clusters of strong positive correlations described in the text.

We constructed co-expression matrices to explore patterns of co-regulation among genes. There were a greater number of significant correlations in the daily warming treatment than the heat shock treatment, 295 and 244, respectively (Figure 5; Table 5). Positive correlations in expression levels among genes were more prevalent under daily warming conditions than heat shock, while the number of negative correlations in expression levels was similar between treatments (Table 5). Correlations in the daily warming treatment were stronger on average than correlations in the heat shock treatment, both more positive and more negative (Table 5). In the daily warming treatment, there were three major clusters of strong positive correlations: 1) between molecular chaperones, 2) molecular chaperones and a mixed group of metabolic genes, transporters, and transcription/translation regulators, and 3) the group of metabolic genes, transporters and transcription/translation regulators with themselves. In the heat shock treatment, cluster 1was maintained (between molecular chaperones) while cluster 2 was reduced. In the daily warming treatment, there were 3 genes with strong negative correlations with many other genes: *protein henna, apoptosis inhibitor,* and *GST*. However, the sign of the correlations between protein henna and the molecular chaperones changed under heat shock conditions. *Protein henna* was positively correlated with molecular chaperones under heat shock conditions but negatively correlated with the same genes under daily warming.

**TABLE 5:** Significant gene correlations in each warming treatment.

Treatment	Mean positive	SE positive	N positive	Mean negative	SE negative	N negative	Total significant
Daily Warming	0.552	0.013	198	−0.349	0.018	97	295
Heat Shock	0.390	0.020	152	−0.254	0.020	92	244

## Discussion

Accurately predicting the future of ectotherms to warming requires studies that include populations with distinct thermal histories to understand variations in the physiological response to warming. In the current study, we tested the molecular physiology responses of three populations of *D. gilvipes* from different eco-regions (mountain, valley, and coast) to different heat exposures. We found population-specific responses under the control and daily warming conditions, while responses to heat shock were similar across populations. In addition, underlying gene expression patterns in the daily warming and heat shock treatments were different.

### Mild temperatures differentiate populations, but extremes connect them

Under control conditions, the three populations had distinct expression profiles. As temperature-sensitive biomarkers, heat shock proteins can signal current stress or the effects of thermal history (Feder and Hoffman, 1999; Dahlhoff, 2004; King and Macrae, 2015). Given that these animals were not exposed to temperatures we expected to induce stress, differences in constitutive expression of thermally sensitive transcripts support our hypothesis that the populations experienced distinct thermal histories (Bowler 2005; King and Macrae 2015).

Thermal history likely also contributed to and magnified the gene expression responses in the daily warming treatment. There was a 5°C warming trend at the Sagehen site beginning 12 days before the experiment was performed, while the other sites stayed within 2.5°C. This rapid warming may have already induced a warming response that was magnified during an otherwise mild temperature increase that resulted in molecular chaperone expression under daily warming conditions that was greater than or equal to the expression of those genes under heat shock (Figures 3, 4). This difference between the response to daily warming at Sagehen and the other sites may also be related to differences in the thermal sensitivity of gene expression. The average Δ expression at Sagehen was two times that of other populations after a similar increase in temperature. Big Creek and Angelo are generally warmer than Sagehen throughout the year. This supports our hypothesis that warm-adapted populations would be less thermally sensitive and exhibit a more muted expression response to warming. Similarly, the common killifish, *Fundulus heteroclitus*, from warm-acclimated populations showed a muted response in the expression of several hsp70 isoforms in response to high temperatures when compared with cold-adapted populations (Fangue et al., 2006). Muted responses in other physiological traits have also been measured in response to warmer thermal histories (Seebacher et al., 2015; Tanner et al., 2019).

Under heat shock conditions, there were no differences between the populations. Potentially, 30°C is approaching a sub-lethal thermal limit and the response to such temperatures is canalized evolutionarily. The CTmax for several North American aquatic insects, including caddisflies, is near 30°C (Houghton et al., 2014; Houghton and Shoup, 2014; Hotaling et al., 2020). Hotaling et al. (2020) also found no transcriptomic grouping by population in high altitude stoneflies exposed to their CTmax. This evidence does not support our hypothesis that we would see a muted thermal response in populations that are already warmer since all populations responded similarly. This contrasts with the differences in thermal sensitivity we saw in the daily stream warming treatment. Extreme warming (rate and maximum temperature achieved) may trigger a consistent species-level response that supersedes differences in thermal history.

### Population-specific responses may mask the effects of seasonal acclimatization

We repeated our experiment at Angelo 1 month later in the summer to assess the effect of a warmer thermal history on the same population’s gene expression response. The late samples had lower expression of molecular chaperones than the early samples, especially in the control and daily warming treatments (Figure 2, Supplementary Figure S4). This may be evidence of the effect of thermal history on thermal sensitivity, an effect of developmental stage of the individual caddisflies, or some combination of the two. Several individuals observed in the creek on the experiment day were older but were not included in the experiment. The month between the two trials at Angelo represented a large number of the degree-heating days for emergence. By late June, most caddisflies near our study site on the Eel River will have entered prepupal diapause (Hannaford, 1998; Resh et al., 2011). Similar gene expression effects have been measured in walleye maintained in warming regions of Lake Manitoba for a short or long portion of the summer (Jeffrey et al., 2020). Fish held in the lake until later in the summer had increased expression of molecular chaperones. Though the directionality was reversed in our experiment, it is clear that seasonal acclimation can change gene expression patterns.

The main difference between the early and late experiments is that the later time point had lower values on PC1, which were driven by expression of *apoptosis inhibitor* and *circadian clock protein* (Figure 2, Supplementary Figure S4)*.* This circadian-clock protein, named *daywake* in *Drosophila melanogaster*, was primarily expressed in individuals from Angelo. Only one individual from elsewhere, Sagehen, expressed this gene. In *D. melanogaster*, *daywake* acts as a behavioral thermometer that promotes daytime sleep under warm conditions to avoid heat damage (Yang and Edery, 2019). Higher expression of *daywake* in the later Angelo samples would indicate warmer days, which matches the actual thermal history at the site. However, we would expect to see this gene expressed more widely at Sagehen after the warming trend. Surprisingly, the late Angelo samples were more similar to the earlier Angelo samples and even further differentiated than the other groups from the Sagehen samples in every treatment (Figure 2). This indicates some combination of population-specific and temperature-specific responses occurring under natural conditions.

### Differences between mild and extreme warming are greatest in a subset of genes

The differences we expected to see between the daily warming treatment and the heat shock treatment were only apparent relative to control expression (Figures 3, 4). Even then, the differences were gene-and population-specific. Consistently, the heat shock proteins were more strongly induced in the heat shock treatment at all sites except Sagehen. A large body of research supports our findings that heat shock protein expression increases with higher temperature exposure (Feder and Hoffman, 1999; King and Macrae, 2015; Somero, 2020).

Co-expression patterns between genes also changed between warming treatments suggesting that it is important to focus on the relationships between genes in addition to individual genes. Under daily warming conditions, protein henna, an amino acid metabolism gene, was negatively correlated with molecular chaperones, suggesting that the importance of protein protection superseded energy production. However, the correlation changed sign under the heat shock conditions. This may indicate that our heat shock treatment was so stressful that energy production and protein homeostasis both needed to increase simultaneously. Similarly, Dong and Zhang (2016) also found that molecular chaperones, specifically *hsp70,* were only positively correlated with metabolic genes under extreme heat conditions. Some other changes in co-expression between treatments signaled that some macromolecules were not further protected under extreme heat. Under daily warming conditions, *trehalose transporter* was positively correlated with many genes but negatively correlated with them under heat shock. Trehalose has multiple protective roles during heat stress, such as stabilizing membranes and preventing further unfolding of proteins (Ebner et al., 2019; Somero, 2020). Its decrease in relation to other molecular chaperones at very high temperatures may highlight a change in exactly which molecules are being protected (Ebner et al., 2019).

### Importance of individual and environmental variation

We studied wild-caught and naturally acclimatized individuals, thus incorporating ecologically relevant impacts of thermal history. In addition, we used a warming treatment that mirrored natural increases in water temperature throughout the day. Maintenance of population-specific thermal history allowed us to measure the baseline expression levels of each population more accurately than a laboratory study could. For example, we can contrast the effects of the warming trend that occurred before sampling at Sagehen with stable temperatures before early sampling at Angelo and Big Creek.

However, ecological realism comes at the cost of control and standardization; and in our study, undetected differences in developmental stage, body condition, or sex may have influenced gene expression patterns. We collected individuals in the final larval instar, indicated by case-building materials in this species (Hannaford, 1998; Holomuzki et al., 2013), but we do not know the individual’s age or proximity to pupation. Warming is known to increase developmental rate and has even been seen to drive pupation and emergence during thermal stress experiments in stoneflies (Hannaford, 1998; Hotaling et al., 2020). Though the differences in developmental state were likely products of degree heating days at each site, we must consider that the warming treatment itself may have had an effect.

Studies of ecologically relevant stress must also consider the timing, intensity, duration, and frequency of the stress. Our study matches the ecological timing of stress to the later larval period and used a relevant duration and two relevant warming intensities (current and future), but for only one cycle. In nature, both warming and cooling on a diel cycle contribute to population acclimatization. Under future warming scenarios, caddisflies may experience warming to 30°C repeatedly with carryover effects each day that influence gene expression and phenotypes that may take longer to appear. These carryover effects may be detrimental such as reduced growth and faster development, resulting in small individuals that are phenologically mismatched, or positive effects such as rapid heat hardening that prepare and protect them from the effects of future thermal stress (Bowler, 2005; Bergmann et al., 2010; Verberk and Calosi, 2012; King and Macrae, 2015; Bernhardt et al., 2020; Hotaling et al., 2020). Field-acclimatized gene expression in response to warming over 1 day is a critical snapshot of the processes that underlie the whole organism response. Still, it is only part of the story that will help us understand the future of the species. Aquatic invertebrates in California face a warmer future due to climate change induced droughts and surface water warming (Null et al., 2013; Swain et al., 2014; Diffenbaugh et al., 2015). Using physiology to understand the effects of warming may help us understand which populations are likely to persist or not.

The present study investigated the field-acclimatized transcriptional response to two warming regimes in the three populations of the larval caddisfly *Dicosmoecus gilvipes* from three eco-regions*.* We found that gene expression of populations from different eco-regions differ in cool control and mild warming scenarios, but not under extreme warming. Populations from warmer eco-regions showed evidence of decreased thermal sensitivity under mild warming conditions. Co-expression between genes should be considered to understand the interactions between molecular processes affected by warming. Our results highlight the importance and limitations of measuring the stress response of wild-caught organisms in their natural environment.

## Data Availability

The datasets presented in this study can be found in online repositories. The names of the repository/repositories and accession number(s) can be found below: The RNA-Seq shotgun assembly can be accessed at: https://www.ncbi.nlm.nih.gov/nuccore/GJZL00000000.1/; The unmodified eXpress output data (library) can be accessed at: https://www.ncbi.nlm.nih.gov/geo/query/acc.cgi?acc=GSE206349; The data generated specifically for this manuscript and from which the conclusions are drawn have been submitted to Dryad : https://doi.org/10.6078/D1T41G.
