# Supplementary material for: Mild temperatures differentiate while extreme temperatures unify gene expression profiles among populations of Dicosmoecus gilvipes in California
Source: Front Physiol. 2022 Oct 5;13:990390. doi: 10.3389/fphys.2022.990390 (PMC9581119; doi:10.3389/fphys.2022.990390)
Supplement: Supplementary file 1 [file DataSheet1.pdf]

Supplementary Document to *Mild temperatures differentiate while extreme temperatures unify gene expression profiles among populations of Dicosmoecus gilvipes in California.* **E.E. King and J.H. Stillman. DOI: 10.3389/fphys.2022.990390**

Contents

Pg 1-9: Methods used to generate a de-novo transcriptome for the caddisfly *Dicosmoecus gilvipes,* and description of transcripts whose expression changed with laboratory exposure to warmer temperatures, including genes selected for assay of field-acclimatized populations using NanoString, as cited in the main text of the manuscript.

Pg 10-17: Supplementary figures and tables for the among-population analyses of gene expression profiles as cited in the main text of the manuscript.

Specimen sampling

Dicosmoecus gilvipes were collected from the surface of stones by hand on 19 and 20 July 2012 in the South Fork of the Eel River, 50-200m downstream of the Elder Creek confluence within the University of California Angelo Coast Range Reserve (http://angelo.berkeley.edu; 39°43'53.34"N; 123°38'41.92"W). Specimens were 5th stage instar larvae within their stone casing (staging was based on casing construction, as 5th instar larvae use minerals instead of plant material used in earlier stages (Limm and Power, 2011). Specimens were transported live to the laboratory within 24h of collection and held under common garden conditions at 11°C for two weeks before being transferred to tanks for different temperature conditions. Tank temperatures were thermally ramped over 4 days to temperatures ranging from 15.5°C to 28.6°C at which time they were held for 24 hours prior to sampling. Transcriptomic (RNA-seq) analyses were based on n=32 specimens, with n=8 per temperature treatment (see below table).

Temperature °C	# specimens in RNA-pool	# libraries made	Pool ID	# libraries passing filtration
15.5	8	5	Di_A	5
20	8	5	Di_B	5
25	8	5	Di_C	5
28.6	8	5	Di_D	5

RNA-seq Methods: Library Construction, Sequencing and Bioinformatics

Animals were flash-frozen in liquid nitrogen and the thorax was dissected on dry ice and placed frozen into Tri Reagent (MRC). The thorax was used to maximize muscle tissue and minimize digestive tract symbiont contamination. Thorax tissue was homogenized under liquid N_2 using a TissueLyser II (Qiagen 85300) for 5-10 sec and RNA was extracted using the manufacturer's polysaccharide/proteoglycan removal modified protocol, using BCP and High Salt Precipitation Solution (MRC). Purified RNA was assessed for quantity and quality using a Bioanalyzer (Agilent) and only samples with little to no degradation and adequate concentration were used in subsequent steps. For each exposure temperature, equal amounts of total RNA from each individual were used to make n=5 independent pooled RNA samples. Those n=5 pooled RNA samples were used to make RNAseq libraries following the Stillman laboratory's modifications of the Illumina Tru-Seq RNA v2 kit (Stillman et al 2020). The pooled RNA allowed best sampling of mean levels of gene expression from a greater number of individuals, and to remove sources of technical variation from the ability to detect changes in gene expression.

Libraries size and concentration was determined on the Bioanalyzer using the high sensitivity DNA chip. Median library size was 400-450 bp across samples. Libraries were multiplexed and sequenced (100 bp paired-end) on the Illumina HighSeq 2000 platform at the Vincent J. Coates sequencing laboratory at UC Berkeley

(http://qb3.berkeley.edu/qb3/gsl/index.cfm). Following qPCR to ensure equal concentrations of each sample, samples were multiplexed on a single sequencing lane.

In what follows we briefly summarize our bioinformatics pipeline. All scripts can be found on the GitHub page for Dr. Scott Fay (https://github.com/safay/RNA_seq/tree/master/blacklight_pipeline). RNAseq data were analyzed on the Pittsburg Supercomputer Center Blacklight. The mean number of processed reads in each library was $11.8 \pm 2.3 \times 10^6$ (mean ± SD). To prepare libraries for analysis, sequences were trimmed to remove Illumina adaptors (stringency = 1) and bases with a Phred quality score under 20 using Trim_Galore! (V 0.3.0). Trimmed reads were 32.0 ± 1.2 % of the total, 11.0 ± 1.8 % of which were quality trimmed, and 0.51 ± 0.02 % of the bases were trimmed. FLASH (V 1.2.8) was used to join overlapping paired end reads in order to make longer reads for *de novo* assembly and to eliminate artificial double counting of mapped read overlap regions. The number of reads processed by FLASH was $10.4 \pm 2.0 \times 10^6$, with 37.8 ± 5.5% of the reads combined, and 11.6 ±1.0% of sequence pairs removed. *De novo* transcriptome assembly was performed using Trinity (V r2013-11-10) (http://trinityrnaseq.sourceforge.net/) (Grabherr et al., 2011). Trinity was used to conduct the assembly using a minimum kmer coverage = 2. The assembled transcriptome contained n=135858 contigs. This Transcriptome Shotgun Assembly project has been deposited at DDBJ/EMBL/GenBank under the accession GJZL00000000. The version described in this paper is the first version, GJZL01000000. The transcriptome was annotated against the SwissProt database using Trinotate (V 1.1) , with BLASTx of nucleotide sequences (resulting in n=37464 annotated contigs), and BLASTp (n=31743 annotated) and PFAM (n=30225 annotated) analyses of TransDecoder (V 2.0) produced protein sequences. Gene ontology annotation was found for n=35024 contigs.

Libraries were mapped to the *de novo* transcriptome for that species using Bowtie2 (V 2.0.6) and counted using eXpress (V 1.5.1) (http://bio.math.berkeley.edu/eXpress/), a read mapper that probabilistically assigns reads that ambiguously map to multiple loci, thus minimizing issues arising from redundant putative transcripts in *de novo* assembled transcriptomes (Roberts & Pachter, 2013). The unmodified eXpress output data are available at the NCBI Gene Expression Omnibus (NCBI GEO) under accession GSE206349 (https://www.ncbi.nlm.nih.gov/geo/query/acc.cgi?acc=GSE206349). Library size (# of mapped reads) was compared visually using boxplots to ensure that there was no library size variation. On average 93.8 ± 1.0 % of reads in each library mapped to the transcriptome.

Statistical analysis of differential gene expression was performed in R using the Bioconductor package EdgeR (V 1.2.4) (http://www.bioconductor.org/packages/release/bioc/html/edgeR.html) (Robinson et al, 2010) to identify genes with expression that varied across temperature. We used likelihood ratio tests with false-discovery rate correction to identify genes that had differential expression between any two acclimation treatments within a given species. Transcripts with expression of less than 2 reads/million mapped in less than 4 samples were removed from the analysis, which resulted in the retention of n=30092 transcripts in the analysis. Transcripts with a false-discovery rate corrected P-value of less than 0.05 and a fold-change ≥ 4 (for up-regulation) or ≤ 0.25 (for down-regulation) were identified as statistically differentially expressed genes (DEGs) in each LRT, which yielded a set of n=2586 DEGs. Differentially expressed transcripts were log2base transformed and median-centered based on the global within-species median. That set of transformed data is available at the NCBI GEO under accession GSE206349. Expression data were log2base transformed and median-centered based on the global within-species median.

Expression data were filtered again (MaxVal-MinVal ≥ 1.0), resulting in a final set of n=934 DEGs, and k-mean clustered (using default parameters) in Cluster (V 3.0; http://bonsai.hgc.jp/~mdehoon/software/cluster/software.htm) and visualized using TreeView (http://jtreeview.sourceforge.net). Those clusters were visualized using TreeView (http://jtreeview.sourceforge.net) to determine the minimum number of k-means clusters that fully described the data. All subsequent data analysis of differentially expressed genes was performed in R.

Gene Expression Clusters:

Expression patterns for *D. gilvipes* are presented in the 8 k-means clusters named alphabetically "A" through "H" (Fig. S1; Table S1). Three of these clusters (A, B, and C) represented genes with a general pattern of down-regulation with increasing temperature, with cluster B showing the greatest downregulation at 30°C (approximately -2 log_2-fold change on average; Fig. S1). All three of these clusters had a high representation of genes related to protein synthesis/degradation (Table S1). In addition, Cluster A had many genes related to transcription and RNA/DNA binding, Cluster B contained several genes related to immune/stress responses, oxidative metabolism, and storage, and Cluster C included many genes related to extracellular/cell-cell interaction/cuticular function and lipid modification (Table S1).

Three of *the D. gilvipes* clusters (D, E, and F) were associated with general increases in gene expression with rising temperature, particularly at 30°C. Clusters E demonstrated the strongest induction at 30°C, with a mean four-fold log_2 increase in expression (Fig. S1). Cluster D was dominated by immune/stress response genes, including heat-shock proteins, as well as cellular chaperones typically unassociated with the stress response (Table S1). Cluster D also

contained a high proportion of genes related to cell cycle/development and transcription and DNA/RNA binding. Cluster E had a high representation of genes related to the cell cycle/development as well as the immune/stress response (Table S1). Cluster F had low and relatively even representation of genes across functional categories, but had the functions with the highest representation were related to amino acid metabolism, immune/stress response, and transcription and DNA/RNA binding (Table S1).

Two of the *D. gilvipes* clusters (G and H) had no strong pattern of change with temperature (Fig. S1). Cluster G contained only features in the "other" functional category, and cluster H had low and relatively even representation across functional categories, with no more than three features occurring for any given function (Table S1).

Table S1. Statistics on clustering of differentially expressed genes.

	Cluster								
	A	B	C	D	E	F	G	H	Total
Total No. DE features	139	163	108	264	25	136	22	77	934
No. DE features annotated	60	74	57	132	19	46	5	18	411
No. unique annotations*	47	55	42	88	10	39	3	17	242
amino acid metabolism	1	2	2	2	0	8	0	3	18
biological rhythms	1	4	0	2	0	1	0	1	9
carbohydrate metabolism	2	2	2	2	0	3	0	1	12
cell cycle/development	5	0	2	24	9	4	0	1	45
chaperones (not stress related)	0	0	0	15	2	0	0	0	17
digestion	1	3	2	0	0	0	0	2	8
extracellular/cell-cell interaction/cuticle	7	13	0	1	0	0	0	1	22
immune/stress response	3	5	14	39	7	5	0	3	76
lipid modification	4	11	1	1	0	3	0	2	22
membrane structure	0	0	0	0	0	1	0	0	1
muscle function	0	3	2	0	0	2	0	0	7
neuronal function	0	1	1	3	0	0	0	0	4
osmotic/ionic regulation	0	0	0	2	0	0	0	1	3
oxidative metabolism	2	8	6	6	0	2	0	1	25
protein synthesis/degradation	10	13	5	6	0	1	0	1	36
storage	0	4	8	0	0	0	0	0	12
cytoskeleton	1	0	2	2	0	1	0	0	6
transcription, DNA/RNA binding	11	1	0	17	0	5	0	0	34
transport	7	0	1	1	0	1	0	0	10
other	5	4	9	10	1	9	5	1	44

* unique annotations in a cluster are specific to that cluster

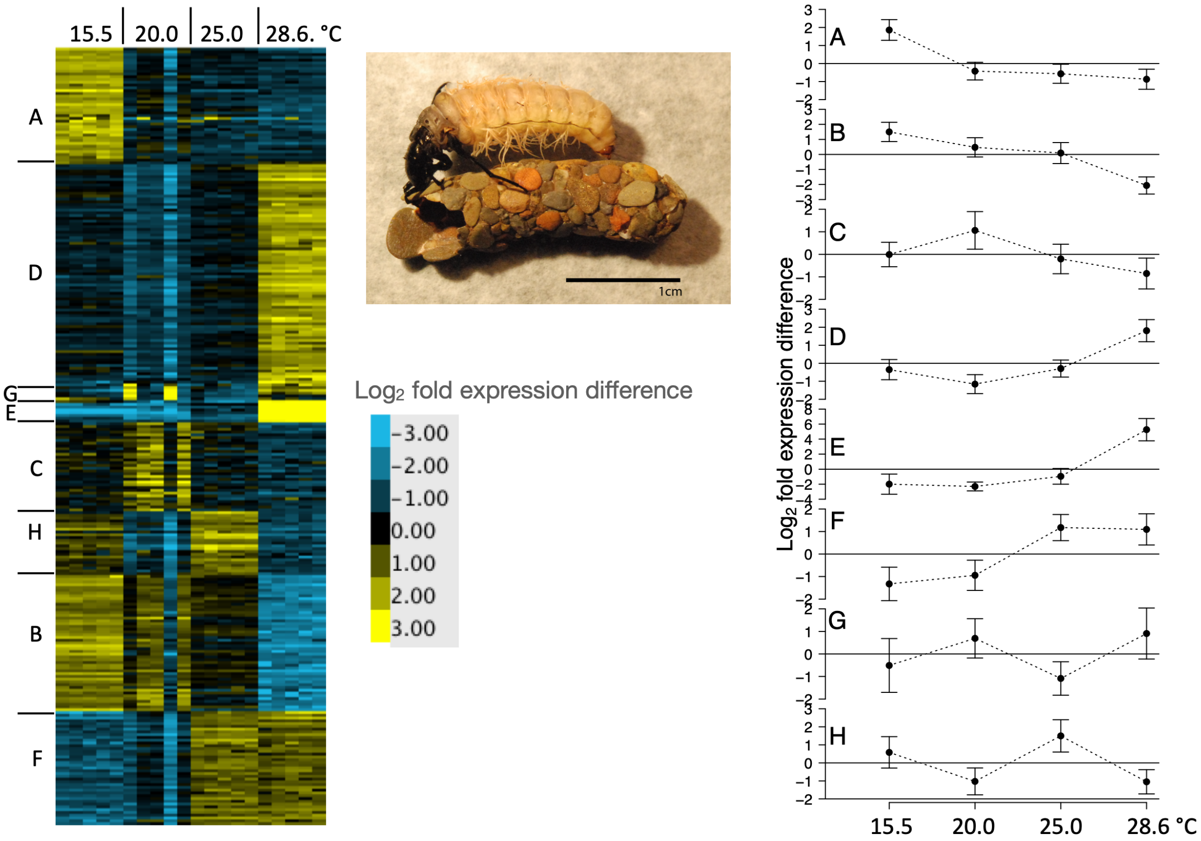

Figure S1. Transcriptome profiles of *Dicosmoecus gilvipes* acclimated to different temperatures. Left, heat map of all data organized into *k*-Means clusters. Center, photo of *D. gilvipes* 5th instar larva, with stony case. Right, mean $\log_2$-fold median-centered normalized FPKM expression difference ± 1 s.d. within *k*-Means clusters (left).

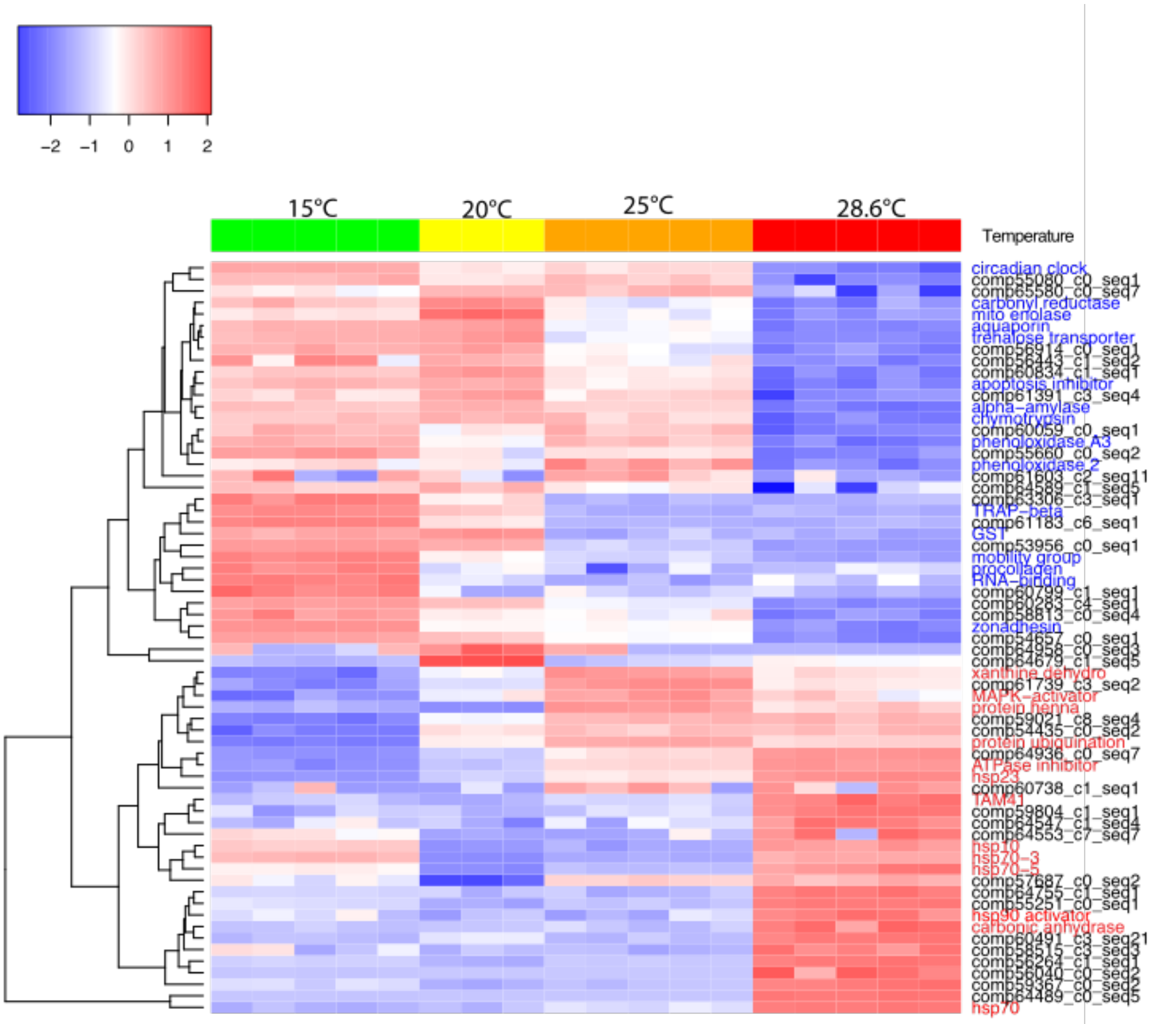

Figure S2. RNA-Seq data only for the genes used in the current study, organized by hierarchical clustering. The text colors represent log2 fold difference in expression, and indicate if we expected an increase (red) or decrease (blue) in transcript abundance with temperature in the current study. Two samples were removed from the 20°C group because of divergent expression patterns (see Fig. S1).

Bibliography

Grabherr MG, Haas BJ, Yassour M, Levin JZ, Thompson DA, Amit I, Adiconis X, Fan L, Raychowdhury R, Zeng Q, Chen Z, Mauceli E, Hacohen N, Gnirke A, Rhind N, di Palma F, Birren BW, Nusbaum C, Lindblad-Toh K, Friedman N and Regev A (2011) Full-length transcrip- tome assembly from RNA-Seq data without a reference genome. Nature Biotechnology 29, 644–652.

Limm MP and Power M (2011) The caddisfly *Dicosmoecus gilvipes*: Making a case for a functional role Journal of the North American Benthological Society 30(2):485-492 DOI:10.1899/10-028.1

Roberts A and Pachter L (2013) Streaming fragment assignment for real-time analysis of sequencing experiments. Nature Methods 10, 71–73.

Robinson MD, McCarthy DJ and Smyth GK (2010) Edger: a Bioconductor package for differential expression analysis of digital gene expression data. Bioinformatics (Oxford, England) 26, 139–140.

Stillman JH, Fay SA, Ahmad SM, Swiney KM, Foy RJ (2020). Transcriptomic response to decreased pH in adult, larval and juvenile red king crab, *Paralithodes camtschaticus*, and interactive effects of pH and temperature on juveniles. Journal of the Marine Biological Association of the United Kingdom 1–15. https://doi.org/10.1017/ S002531541900119X

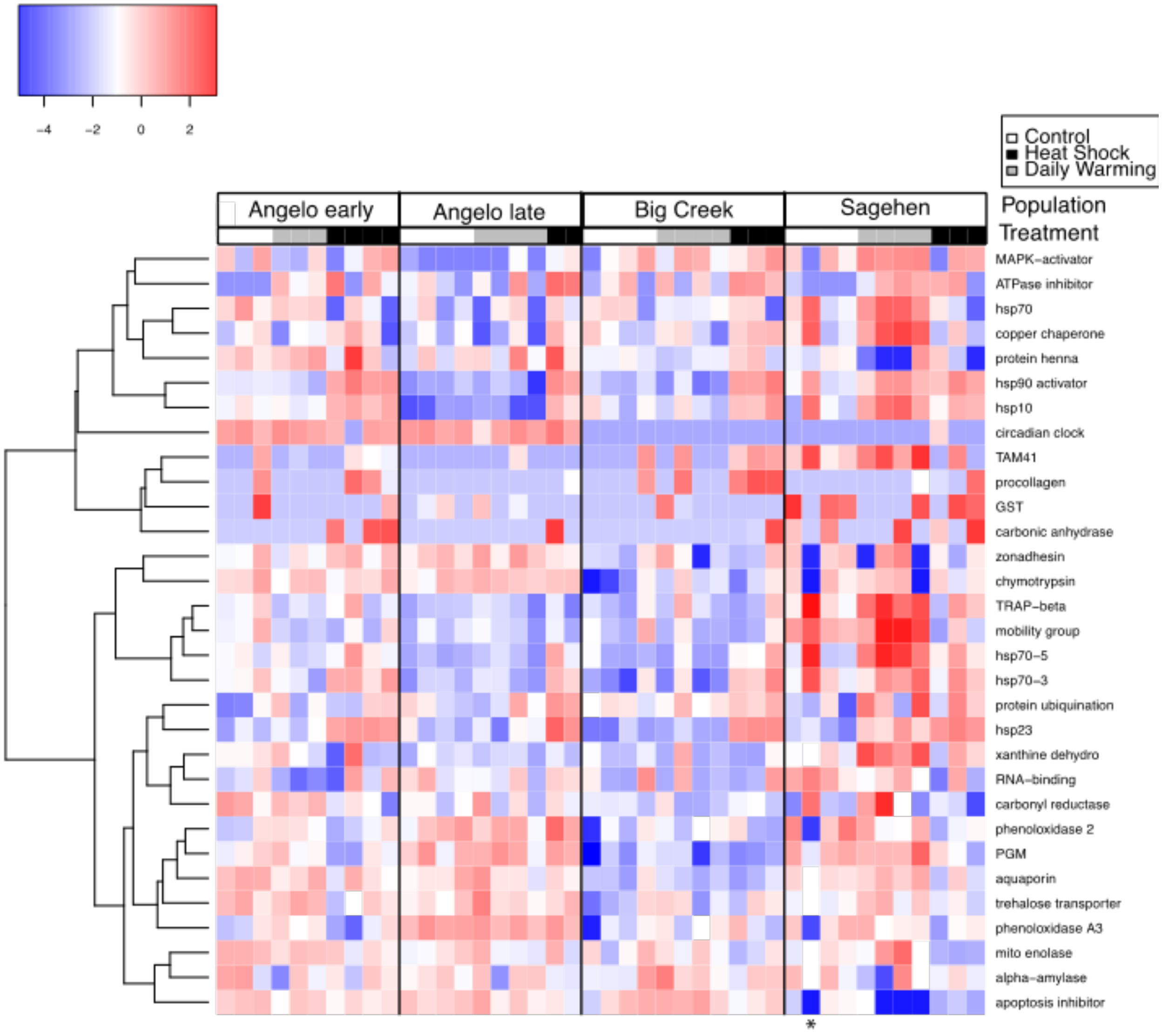

Figure S3. Heatmap of all genes and treatments, without any induction calculations. Genes are arranged in rows and grouped by similarity of expression value (dendrogram). Each column is an individual caddisfly, labeled by its warming treatment. White bars represent individuals in the cool control treatment. Black and grey bars correspond to the daily warming treatment and heat shock, respectively. The colors of the heat map cells represent the magnitude and direction of the change in expression, scaled and centered by row. Asterisk represents the individual left out of the PCA analysis.

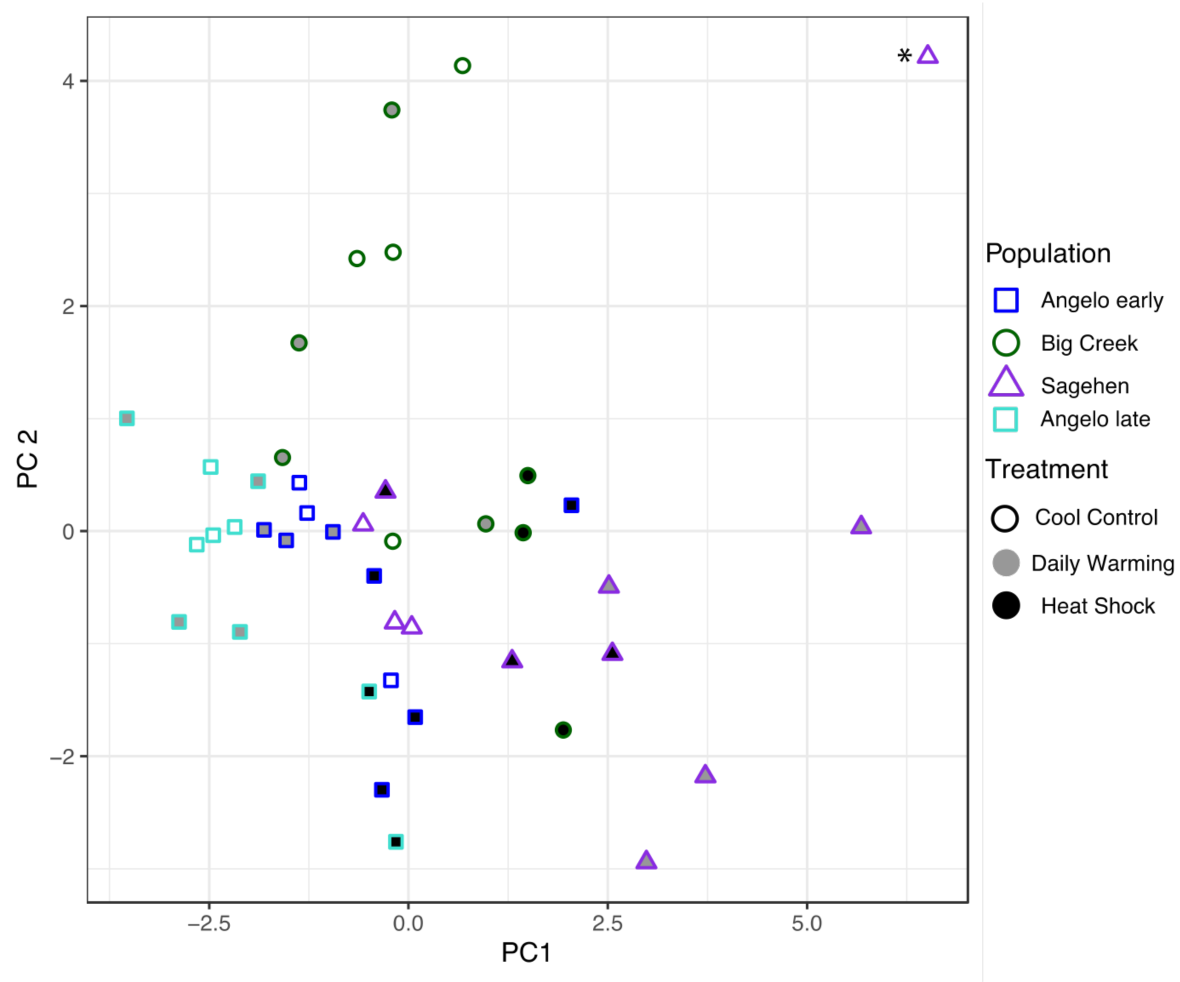

Figure S4. Population and treatment differentiation along principal components 1 and 2. This figure and analysis are identical to Fig 2, except that it includes one outlier point from Sagehen control, represented by the asterisk. Data represent PC scores for all population and treatment combinations. Symbol shape represents population of origin, fill color represents the treatment.

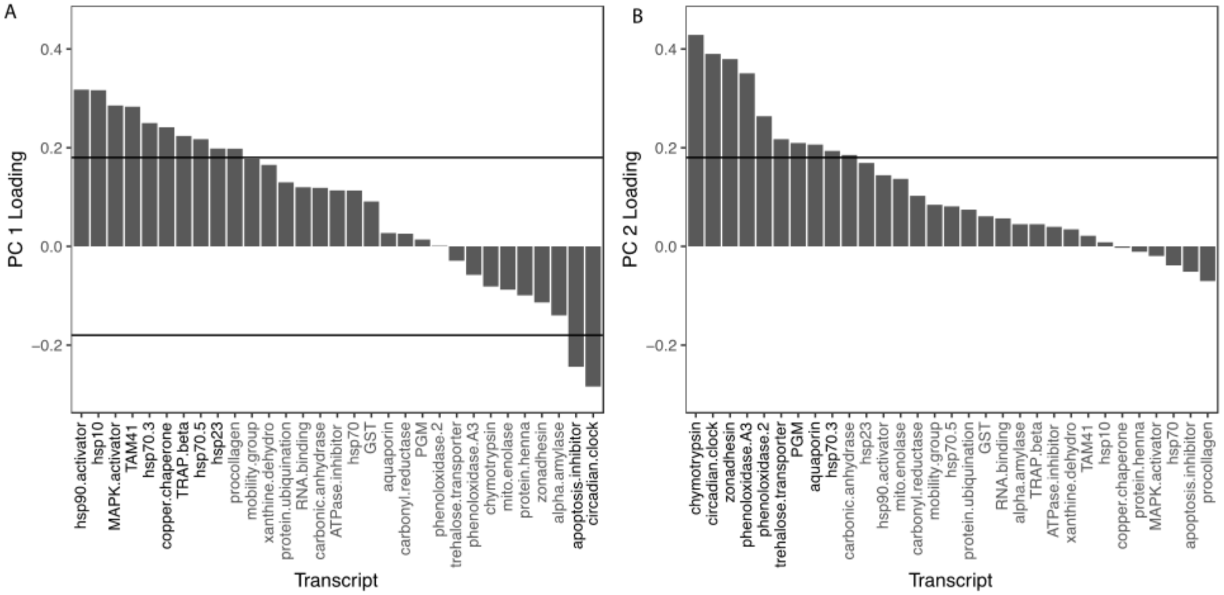

Figure S5. Principal component loadings for **A)** PC 1 **B)** PC 2. Horizontal lines indicate significance level. Transcripts with significant loadings are in black. All others are in gray.

Table S2. NanoString target and reference genes

**Target Transcripts**					
**Abbreviation**	**Full Transcript Name**	**ContigID**	**UniProtID**	**Function**	**Expected Heat Δ**
alpha-amylase	Alpha-amylase A	comp57363_c0_seq1	P08144	carbohydrate metabolism	down
apoptosis inhibitor	Apoptosis inhibitor	comp58747_c0_seq1	P41436	apoptosis inhibition	down
aquaporin	Aquaporin AQPAe.a	comp64170_c1_seq1	Q9NHW7	transporter activity	down
ATPase inhibitor	ATPase inhibitor mai-2	comp60498_c0_seq1	A8XZB0	negative regulation of ATPase activity	up
carbonic anhydrase	Carbonic anhydrase 2	comp61667_c0_seq1	Q8UWA5	carbonate dehydratase activity	up
carbonyl reductase	Carbonyl reductase [NADPH]	comp61657_c0_seq4	Q28960	metabolic process	down
chymotrypsin	Chymotrypsin-1	comp55087_c0_seq1	Q27289	digestion	down
circadian clock	Circadian clock-controlled protein	comp57418_c0_seq3	O76879	circadian rhythm	down
copper chaperone	Copper chaperone for superoxide dismutase	comp58560_c3_seq5	Q9JK72	metal ion transport, superoxide radical removal	up
GST	Glutathione S-transferase	comp58392_c0_seq1	P46437	Transferase, antioxidant	down
hsp10	10 kDa heat shock protein, mitochondrial	comp63721_c5_seq1	Q5DC69	protein folding	up
hsp23	Heat shock protein 23	comp54992_c0_seq1	P02516	protein folding	up
hsp70	Heat shock protein 70 B2	comp64140_c1_seq7	P41827	stress response	up
hsp70-3	Heat shock 70 kDa protein cognate 3	comp58617_c2_seq1	P29844	protein folding	up

hsp70-5	Heat shock 70 kDa protein cognate 5	comp63373_c1_seq4	P29845	protein folding	up
hsp90 activator	Activator of 90 kDa heat shock protein ATPase homolog 1	comp63956_c0_seq2	O95433	co-chaperone of hsp90	up
MAPK-activator	Arf-GAP with dual PH domain-containing protein 1	comp62480_c4_seq2	O75689	regulation of GTPase activity	up
mito enolase	Mitochondrial enolase superfamily member 1	comp58198_c0_seq2	Q7L5Y1	amino acid and carbohydrate catabolism	down
mobility group	Mobility group protein 1A	comp38073_c0_seq1	P40622	DNA binding	down
PGM	Phosphoglucomutase	comp63948_c2_seq1	Q7KHA1	glucose metabolic process	down
phenoloxidase 2	Phenoloxidase subunit 2	comp61132_c0_seq1	Q27452	melanin biosynthesis, defense response	down
phenoloxidase A3	Phenoloxidase subunit A3	comp56149_c0_seq1	Q9V521	melanin biosynthesis, defense response	down
procollagen	Procollagen-lysine,2-oxoglutarate 5-dioxygenase 1	comp61977_c0_seq1	P24802	collagen formation	down
protein henna	Protein henna EC	comp64492_c0_seq6	P17276	amino acid metabolism	up
protein ubiquination	E3 ubiquitin-protein ligase RNF139	comp63534_c0_seq2	Q8WU17	protein ubiquination regulation	up
RNA-binding	RNA-binding protein Rsf1	comp55145_c0_seq1	Q24491	gene expression regulation	down
TAM41	Mitochondrial translocator assembly and maintenance protein 41 homolog	comp60449_c0_seq4	Q8INF2	cardiolipin biosynthesis	up
TRAP-beta	Translocon-associated protein subunit beta	comp31633_c0_seq1	P23438	co-translational protein targeting to membrane	down
trehalose transporter	Facilitated trehalose transporter Tret1	comp63446_c0_seq1	A9ZSY3	sugar transport	down

xanthine dehydro	Xanthine dehydrogenase	comp64423_c0_seq2	P08793	xanthine catabolic process	up
zonadhesin	Zonadhesin Flag	comp62844_c0_seq3	O88799	cell adhesion	down
**Reference Transcripts**					
elongation 1B	Elongation factor 1-beta	comp51037_c0_seq1	P29522	Protein biosynthesis	down
troponin	Troponin I	comp62634_c1_seq14	P36188	Actin binding	down
unknown ref	NA	comp60946_c5_seq1	NA	NA	up

Table S3 . Tukey multiple comparison tests. Significance codes for adjusted p-values: 0=***, 0.001=**, 0.01=*, 0.05=., 0.1=NS **A)** Population differentiation PC1 **B)** Population differentiation PC2 **C)** Angelo dates PC1

A)

	P adj
Population	
BigCreek-Angelo	NS
Sagehen-Angelo	***
Sagehen-BigCreek	***
Treatment	
HS-control	*
stream-control	*
stream-HS	NS
Population*Treatment	
Sagehen:stream - Angelo:stream	***
Sagehen:stream - BigCreek:stream	***
Sagehen:stream - BigCreek:control	***
Sagehen:stream - Angelo:control	***
Sagehen:stream - Sagehen:control	***
Sagehen:stream - Angelo:HS	**
Angelo:stream - BigCreek:HS	**
Sagehen:stream - Sagehen:HS	**
BigCreek:stream - BigCreek:HS	**
Angelo:stream - Sagehen:HS	.
BigCreek:HS - BigCreek:control	.
BigCreek:HS - Angelo:control	.
Sagehen:stream - BigCreek:HS	NS
BigCreek:stream - Sagehen:HS	NS
Sagehen:HS - Angelo:control	NS
Sagehen:HS - BigCreek:control	NS
Angelo:stream - Angelo:HS	NS
BigCreek:HS - Sagehen:control	NS
BigCreek:stream - Angelo:HS	NS
Angelo:HS - Angelo:control	NS
Angelo:HS - BigCreek:control	NS
Angelo:stream - Sagehen:control	NS
Sagehen:HS - Sagehen:control	NS
BigCreek:HS - Angelo:HS	NS
BigCreek:stream - Sagehen:control	NS
Sagehen:control - Angelo:control	NS
Sagehen:HS - Angelo:HS	NS
Angelo:stream - BigCreek:control	NS
Sagehen:control - BigCreek:control	NS
Angelo:HS - Sagehen:control	NS
BigCreek:stream - Angelo:stream	NS
Angelo:stream - Angelo:control	NS
Sagehen:HS - BigCreek:HS	NS
BigCreek:stream - BigCreek:control	NS
BigCreek:control - Angelo:control	NS
BigCreek:stream - Angelo:control	NS
Sagehen:control - Angelo:control	NS
Sagehen:HS - Angelo:HS	NS
Angelo:stream - BigCreek:control	NS

B)

	P adj
Population	
BigCreek - Angelo	***
Sagehen - Angelo	NS
Sagehen - BigCreek	***
Treatment	
HS-control	NS
stream-control	NS
stream-HS	NS
Population*Treatment	
Angelo:HS - BigCreek:control	*
Sagehen:stream - BigCreek:control	*
BigCreek:control - Angelo:control	*
Sagehen:control - BigCreek:control	.
BigCreek:stream - Angelo:HS	NS
BigCreek:stream - Angelo:control	NS
Sagehen:stream - BigCreek:stream	NS
BigCreek:stream - Angelo:stream	NS
Sagehen:HS - BigCreek:control	NS
BigCreek:stream - Sagehen:control	NS
BigCreek:stream - Sagehen:HS	NS
BigCreek:HS - BigCreek:control	NS
BigCreek:HS - Angelo:HS	NS
BigCreek:HS - Angelo:control	NS
Angelo:stream - BigCreek:HS	NS
Sagehen:stream - BigCreek:HS	NS
BigCreek:HS - Sagehen:control	NS
BigCreek:stream - BigCreek:HS	NS
Sagehen:HS - Angelo:HS	NS
Sagehen:HS - Angelo:control	NS
Sagehen:HS - BigCreek:HS	NS
BigCreek:stream - BigCreek:control	NS
Angelo:stream - Sagehen:HS	NS
Sagehen:stream - Sagehen:HS	NS
Sagehen:HS - Sagehen:control	NS
Sagehen:stream - Angelo:HS	NS
Sagehen:stream - Angelo:control	NS
Angelo:HS - Sagehen:control	NS
Sagehen:control - Angelo:control	NS
Sagehen:stream - Angelo:stream	NS
Angelo:HS - Angelo:control	NS
Angelo:stream - Angelo:control	NS
Angelo:stream - Sagehen:control	NS
Sagehen:stream - Sagehen:control	NS
Angelo:stream - Angelo:HS	NS

C)

	P adj
Date	
early-late	***
Treatment	
HS-control	***
stream-control	NS
stream-HS	***
Date*Treatment	
late:stream - early:HS	***
early:HS - late:control	***
late:stream - late:HS	**
late:HS - late:control	**
early:stream - early:HS	*
late:stream - early:control	.
late:control - early:control	.
early:HS - early:control	NS
early:stream - late:HS	NS
late:stream - early:stream	NS
early:stream - late:control	NS
late:HS - early:control	NS
early:stream - early:control	NS
late:HS - early:HS	NS
late:stream - late:control	NS
late:stream - early:control	NS
